# Accuracy between prehospital and hospital diagnosis in helicopter emergency medical services and its consequences for trauma care

**DOI:** 10.1007/s00068-024-02505-y

**Published:** 2024-04-02

**Authors:** Martin Müller, Wolf Hautz, Yves Louma, Jürgen Knapp, Beat Schnüriger, Hans-Peter Simmen, Urs Pietsch, Dominik A. Jakob, the Swiss Trauma Board

**Affiliations:** 1 Department of Emergency Medicine, Inselspital, Bern University Hospital, University of Bern, 3010 Bern, Switzerland; 2 Department of Anaesthesiology and Pain Medicine, Inselspital, Bern University Hospital, University of Bern, Bern, Switzerland; 3 Department of Visceral Surgery and Medicine, Inselspital, Bern University Hospital, University of Bern, Bern, Switzerland; 4 Department of Traumatology, University Hospital Zurich, University of Zurich, Ramistrasse 100, 8091 Zurich, Switzerland; 5 Division of Perioperative Intensive Care Medicine, Cantonal Hospital St. Gallen, St. Gallen, Switzerland; 6 Swiss Air-Ambulance, Rega (Rettungsflugwacht/Guarde Aérienne), Zurich, Switzerland

**Keywords:** Prehospital diagnosis, Helicopter emergency medical services, Prehospital interventions

## Abstract

**Purpose:**

For optimal prehospital trauma care, it is essential to adequately recognize potential life-threatening injuries in order to correctly triage patients and to initiate life-saving measures. The aim of the present study was to determine the accuracy of prehospital diagnoses suspected by helicopter emergency medical services (HEMS).

**Methods:**

This retrospective multicenter study included patients from the Swiss Trauma Registry with ISS ≥ 16 or AIS head ≥ 3 transported by Switzerland’s largest HEMS and subsequently admitted to one of twelve Swiss trauma centers from 01/2020 to 12/2020. The primary outcome was the comparison of injuries suspected prehospital with the final diagnoses obtained at the hospital using the abbreviated injury scale (AIS) per body region. As secondary outcomes, prehospital interventions were compared to corresponding relevant diagnoses.

**Results:**

Relevant head trauma was the most commonly injured body region and was identified in 96.3% (95% CI: 92.1%; 98.6%) of the cases prehospital. Relevant injuries to the chest, abdomen, and pelvis were also common but less often identified prehospital [62.7% (95% CI: 54.2%; 70.6%), 45.5% (95% CI: 30.4%; 61.2%), and 61.5% (95% CI: 44.6%; 76.6%)]. Overall, 7 of 95 (7.4%) patients with pneumothorax received a chest decompression and in 22 of 39 (56.4%) patients with an instable pelvic fracture a pelvic binder was applied prehospital.

**Conclusion:**

Approximately half of severe chest, abdominal, and pelvic diagnoses made in hospital went undetected in the challenging prehospital environment. This underlines the difficult circumstances faced by the rescue teams. Potentially life-saving interventions such as prehospital chest decompression and increased use of a pelvic binder were identified as potential improvements to prehospital care.

**Supplementary Information:**

The online version contains supplementary material available at 10.1007/s00068-024-02505-y.

## Introduction

Trauma remains a leading cause of mortality and morbidity, although worldwide, many injuries are declining. In 2019, 8% of all death globally were trauma-related, accounting for approximately six million deaths per year [1, 2]. Similarly, in Switzerland, accidents are among the most common causes of premature mortality and are the second most common reason for hospitalization [3].

Prehospital trauma care is an important cornerstone in the rescue chain. In Switzerland and many other Western countries, helicopter emergency medical services (HEMS) have become a standard element of modern prehospital trauma care [4, 5]. For optimal prehospital care, it is important that potentially life-threatening injuries are recognized so that patients can be appropriately triaged and immediately life-threatening conditions can be treated without delay. Although advanced trauma life support (ATLS) is possible in severe trauma victims during HEMS missions, there are unsolvable technical and other limitations, like limited space, communication difficulties, and in-flight vibration that may complicate optimal patient care compared to ground-based or even in-hospital care [6]. In addition, the rescue teams often have to work in difficult weather conditions or in unsafe environments.

More than 10 years ago, Hasler et al. evaluated the accuracy of prehospital diagnosis in HEMS missions provided by the Swiss Air-Ambulance Rega [7]. This monocenter retrospective study confirmed that the recognition of injured body regions in the prehospital setting is challenging: abdominal, pelvic, spinal, and chest injuries were frequently unrecognized in the prehospital setting. In recent years, HEMS in Switzerland has advanced significantly. Modern medical equipment, larger and better-equipped helicopters, and, above all, further professionalization of HEMS, including specific training, have been achieved [8, 9]. These developments may also have increased the accuracy of prehospital diagnosis.

The present study was conceptualized as a national multicenter study to assess the prehospital diagnostic accuracy in severely injured trauma patients rescued by HEMS [7]. Of particular interest was the identification of frequently unrecognized but potentially life-threatening injuries and their immediate life-saving interventions in the prehospital setting.

## Methods

### Study design and setting

This retrospective diagnostic accuracy study is reported according to the standards for reporting of diagnostic accuracy studies (STARD) 2015 guidelines [10]. Two different data sources were used and merged to obtain the necessary data of patients with severe injury from 01.01.2020 to 31.12.2020 in Switzerland transported with the largest HEMS company Swiss Air Rescue (Rega) and treated in one of twelve Swiss level 1 trauma centers.

The first database used was the Swiss Trauma Registry (STR). All twelve level 1 trauma centers in Switzerland are obliged by law to document adult trauma patients (≥ 16 years) with an ISS ≥ 16 and/or abbreviated injury score (AIS) head ≥ 3 in a national trauma database, the STR [11]. All injuries of a patient are coded using the AIS [12]. AIS codes contained in the STR are specifically assigned by dedicated coding personnel and are not derived from International Classification of Diseases (ICD) codes. Data management and software is being supplied by an independent specialized certified and audited enterprise (Adjumed Services AG©, ISO 9001:2015). Regular quality controls and audits are conducted at the participating trauma centers to ensure good data quality. STR data were used to determine the final diagnoses, which served as the gold standard in comparison to the prehospital suspected diagnoses. The STR data are coded, and each hospital has the key for their patients included in the registry.

Second, we used both the analog and digital mission protocols of Rega to assess prehospital suspected trauma in each body region, in terms of a diagnostic accuracy study of the *index test*. Rega operates around-the-clock physician-staffed for prehospital retrievals, which are primary missions, as well as interfacility transfers, categorized as secondary missions, within Switzerland. Rega conducts an annual total of approximately 16,000 HEMS missions across Switzerland. A Rega HEMS crew comprises a pilot, a paramedic, and a physician. HEMS physicians are required to hold board certification in anesthesiology and certification in prehospital emergency medicine.

### Participants

HEMS missions were identified from the STR, where type of admission is a mandatory variable. Each hospital was contacted for decoding of the patients. STR and Rega data were merged using a unique allocation code and entered anonymously into the analyses.

### Eligibility criteria

All included trauma missions had to fulfil the following eligibility criteria.

Inclusion criteria:

i. Treated in a level 1 trauma center in Switzerland in the study year 2020
ii. ISS ≥ 16 and/or AIS head ≥ 3
iii. HEMS mission with the company Rega

Exclusion criteria from the STR:

i. Age < 16
ii. Isolated burns (including electrical burns), or if the burn is the predominant injury
iii. Patients who arrive at the trauma room without signs of life, when either no or only very limited diagnostic or treatment measures have been taken for them
iv. Suffocation or hanging without additional injuries
v. Drowning victims

as well as

vi. No encoding of STR patient possible
vii. Patients transported with other HEMS provider
viii. No HEMS mission protocol could be found

### Definition of relevant trauma for a body region (reference standard)

R*elevant trauma for a body region* was defined as at least one injury with an AIS score ≥ 3 in the studied body regions diagnosed in hospital. These were the primary outcomes, the reference standards. The following body regions were analyzed: head, face, neck, abdomen, chest, spine, pelvis, lower, and upper extremity as well as external.

As the AIS score lower extremity category includes pelvic bone injury, we excluded those from the AIS lower extremity and created an AIS score for the pelvis (bone injuries). Thus, we obtained for each patient ten binary variables, one for each body region, for the primary outcomes. An AIS score ≥ 3 was considered as severe trauma, and an AIS score of 1 or 2 was defined as mild trauma.

### Further outcomes

As secondary outcomes, we studied the prehospital identification of the following specific and potentially immediately life-threatening injuries: subdural and epidural hematomas, c-spine injuries, pneumothorax including tension pneumothorax, and instable pelvic fractures. The AIS scores used are listed in the supplemental material (Supplement 1).

### Identification of prehospital relevant trauma for a body region (index test)

All Rega missions are documented in a handwritten protocol including a human pictogram in which areas of suspected/identified injuries can be marked. Additionally, the physician in charge has the opportunity to electronically document affected body parts, main diagnosis, vital signs, and actions performed.

Our approach remained consistent across all trauma cases: when identifying suspected body region injuries, we cross-referenced information from (i) the written sections, (ii) the visual documentation in the human pictogram as well as (iii) the electronically stored information. If any of the documentation marked a body region as injured or suspected injured, the body region was coded as *prehospital suspected/identified injured body region*. Thus, we obtained for each body region a binary outcome, the prehospital “*diagnoses.*” The assessor (YL) of the prehospital “diagnoses” (index test) prior to clinical evaluation remained blinded to the clinical diagnosis and its findings while conducting a thorough full-text analysis of the HEMS report.

### Further data collection and extraction

To describe the patient collective and analyze for associations with unrecognized relevant body trauma, the following additional information were extracted from the STR respectively from the mission reports (analog and digital): demographics, rescue characteristics, accident characteristics, first vital signs (prehospital and in-hospital), prehospital measures (defined elsewhere [13]), injury severity, and clinical outcome (Supplement 2).

### Statistical analysis

All statistical analysis was performed with STATA 18.1 (StataCorp, College Station, TX, USA).

For descriptive analysis, the total number with accompanied percentage was used for categorical variables. Continuous variables were presented with median and interquartile range (IQR) or mean and standard deviation (SD), respectively, depending on normality testing (Shapiro Wilk test).

The – *diagt* – command was used to calculate diagnostic accuracy measures, i.e., sensitivity, specificity, negative, and positive predictive value, negative and positive likelihood radio as well as prevalence with 95% CI. The diagnostic accuracy measures were calculated for each body region (prehospital suspected/identified body region trauma vs. clinically diagnosed relevant trauma respectively the secondary outcomes). The percent of identified relevant/non-relevant and any diagnosis in each body region was visually shown with a spider plot respectively shown with a Scatter plot with 95% CI where the CI was calculated using the – *cii proportion* – command.

No missing data of the index or reference test existed as missing coding of an injury was coded as not present. This was a convenience sample for descriptive analysis; thus, no sample size calculation was performed.

## Results

### Study population

**Fig. 1:**
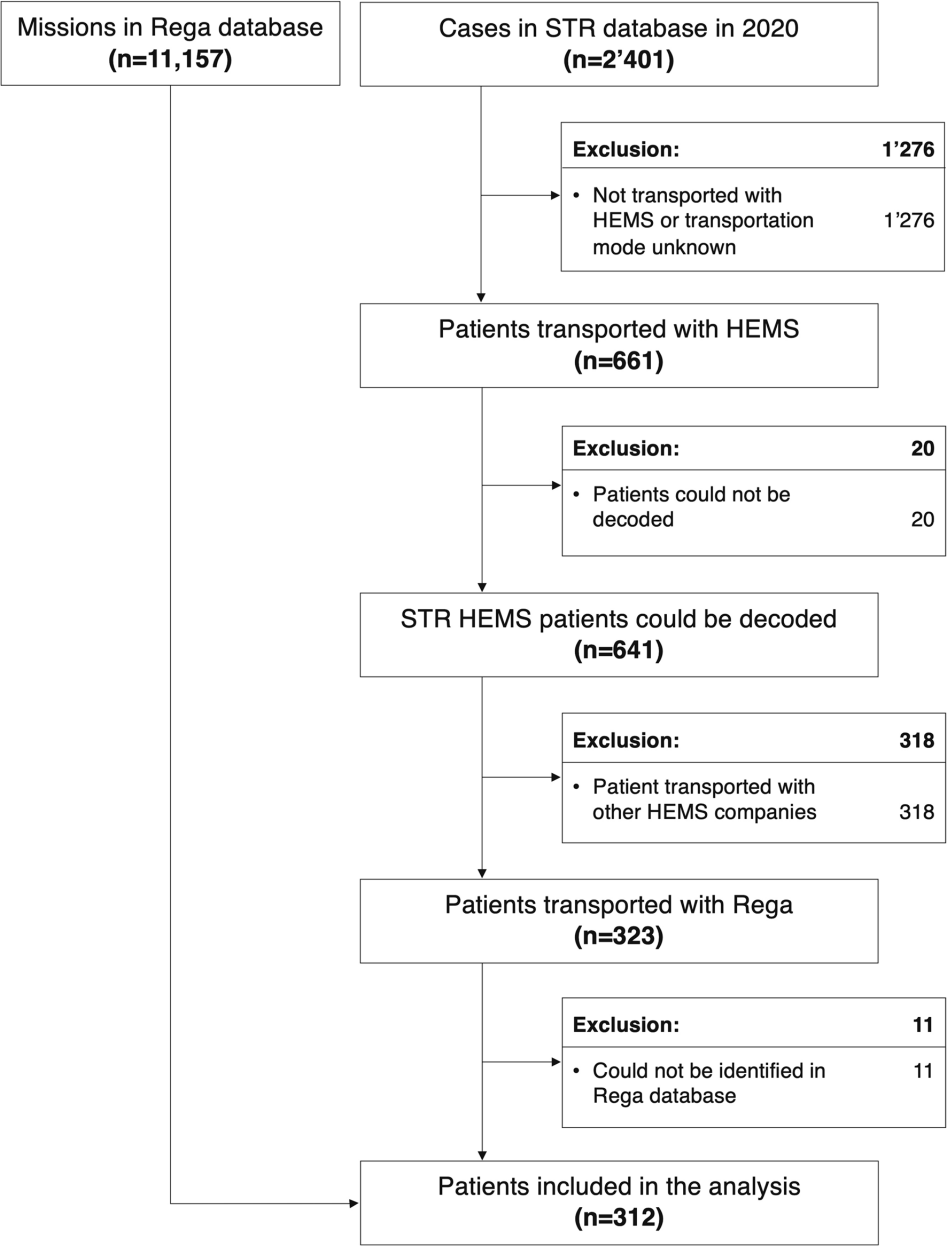
Study flowchart. Abbreviations: HEMS, helicopter emergency medical services; Rega, Swiss largest air rescue company; STR, Swiss Trauma Registry

Of 2401 trauma patients in the STR database and 11,157 REGA missions in 2020, 312 patients were present in both databases and were therefore included in the final analysis (see Fig. 1). A descriptive comparison of included HEMS missions with Ground Emergency Medical Transportation (GEMS) in STR 2020 is shown in Supplement Table 3 with more severe trauma apart from relevant head trauma in HEMS missions.

### Baseline and clinical characteristics

The cohort and prehospital characteristics are shown in Table 1. The median age of participants was 54.5 years (IQR: 34–68), with 76.0% males. Most missions occurred during day time (59.0%), with a median response time of 20 min (IQR 20–39); 15.7% of the patients were rescued with a hoist. Initial vital signs included a median GCS of 14 (IQR 7–15) and a median heart rate of 85 beats per minute (70–102), and a mean systolic blood pressure of 127 mmHg (SD 29).

**Table 1:** Cohort and prehospital characteristics

	*N*	Total	**(***n*** = 312)**
Demographics			
Age [years], median (IQR)	312	54.5	[34; 68]
Gender, *n* (%)	312		
Female		75	[24.0]
Male		237	[76.0]
Rescue characteristics			
Shift, *n* (%)	312		
Day shift (8–16)		184	[59.0]
Late shift (16–24)		95	[30.4]
Night shift (24–8)		33	[10.6]
Duration flight [min], median (IQR)	312	30	[20; 38.5]
Response time [min], median (IQR)	305	20	[15; 27]
On scene time [min], median (IQR)	302	28	[21; 37]
Hoist rescue, median (IQR)	312	49	[15.7]
Prehospital vitals, 1st			
GCS, median (IQR)	312	14	[7; 15]
HR [bpm], median (IQR)	295	85	[70; 103]
SBP [mmHg], mean (SD)	254	127	[29.0]
SpO2 [%], median (IQR)	285	96	[91; 98]
Actions			
Breathing monitoring, *n* (%)	312	304	[97.4]
Basic airway, *n* (%)	312	227	[72.8]
Advanced airway, *n* (%)	312	85	[27.2]
Anesthetics, *n* (%)	312	89	[28.5]
Hemodynamic monitoring, *n* (%)	312	266	[85.3]
Temperature, *n* (%)	312	32	[10.3]
Vascular access (iv/io), *n* (%)	312	296	[94.9]
Catecholamines, *n* (%)	312	40	[12.8]
CPR, *n* (%)	312	5	[1.6]
Cardiac massage, *n* (%)	312	5	[1.6]
PTX decompression, *n* (%)	312	10	[3.2]
Defibrillation, *n* (%)	312	1	[0.3]
Bleeding control, *n* (%)	312	156	[50.0]
Cervical collar, *n* (%)	312	214	[68.6]
Pelvic binder, *n* (%)	312	75	[24.4]
Analgesics, *n* (%)	312	214	[68.6]
Prehospital injury severity estimation			
NACA score, n (%)	312		
Moderate to severe injury/illness		21	[6.7]
Severe injury/illness		160	[51.3]
Life-threatening injury/illness		124	[39.7]
Respiratory and/or circulatory arrest		7	[2.2]
Prehospital injury suspicion			
Head diagnosis, *n* (%)	312	235	[75.3]
Face diagnosis, *n* (%)	312	65	[20.8]
Neck diagnosis, *n* (%)	312	47	[15.1]
Chest diagnosis, *n* (%)	312	118	[37.8]
Abdomen diagnosis, *n* (%)	312	50	[16.0]
Pelvis diagnosis, *n* (%)	312	51	[16.3]
Spine diagnosis, *n* (%)	312	71	[22.8]
Upper extremity diagnosis, *n* (%)	312	78	[25.0]
Lower extremity diagnosis, *n* (%)	312	63	[20.2]
External diagnosis, *n* (%)	312	79	[25.3]

CPR, cardiopulmonary reanimation; *GCS*, Glasgow Coma Scale; *HR*, heart rate; *IQR*, interquartile range; *NACA*, National Advisory Committee for Aeronautics; *PTX*, pneumothorax; *SBP*, systolic blood pressure; *SD*, standard deviation; *SpO2*, oxygen saturation

Prehospital interventions were common, including breathing monitoring (97.4%), basic airway management (72.8%), and vascular access (94.9%). Prehospital advanced airway measures (i.e., intubation, surgical airway, mechanical ventilation) were performed in 27.2%. A pelvic binder was applied in 24.4% of the patients and a cervical collar in 68.6% of the patients.

The most common prehospital injury suspicions/diagnoses were related to the head (75.3%) and chest (37.8%), the less common were neck injuries (15.1%) as well as abdomen (15.0%) and pelvic (16.3%) injuries.

The clinical characteristics and diagnosed injuries are shown in Table 2. The median ISS was 22 (IQR: 17 to 29). Relevant body trauma (AIS ≥ 3) ranged from external (0.3%), lower extremity injuries (1.0%), and neck injuries (2.6%) to 51.6% head and 45.5% chest trauma.

**Table 2:** Clinical characteristics

	*N*	Total	(*n* = 312)
Vitals, 1st clinical			
SBP [mmHg], mean (SD)	312	130.8	[27.9]
HR [bpm], median (IQR)	312	86	[72; 98]
GCS, median (IQR)	301	14	[3; 15]
Respiratory rate [/min], median (IQR)	199	17	[14; 21]
Temperature [°C], median (IQR)	260	36.4	[35.9; 36.8]
SpO2 [%], median (IQR)	261	98	[95; 100]
Injury severity			
ISS, median (IQR)	308	22	[17; 29]
Relevant body trauma			
Head, *n* (%)	312	161	[51.6]
Face, *n* (%)	312	11	[3.5]
Neck, *n* (%)	312	8	[2.6]
Thorax, *n* (%)	312	142	[45.5]
Abdomen, *n* (%)	312	44	[14.1]
Pelvis, *n* (%)	312	39	[12.5]
Spine, *n* (%)	312	60	[19.2]
Upper extr., *n* (%)	312	3	[1.0]
Lower extr., *n* (%)	312	38	[12.2]
External, *n* (%)	312	1	[0.3]
Specific conditions			
SDH,* n* (%)	312	83	[26.6]
EDH, *n* (%)	312	17	[5.4]
C-spine fracture/ligament injury, *n* (%)	312	18	[5.8]
PTX,* n* (%)	312	95	[30.4]
Tension PTX, *n* (%)	312	1	[0.3]
IPV, *n* (%)	312	39	[12.5]
Outcome			
Duration of hospitalization [days], median (IQR)	311	9	[5; 16]
Survival hospitalization, *n* (%)	311	266	[85.5]
Mortality 28 days, *n* (%)	269	49	[18.2]

*EDH*, epidural hematoma; *IQR*, interquartile range; *IPV*, instable pelvis fracture; *ISS*, injury severity score; *PTX*, pneumothorax; *SD*, standard deviation; *SDH*, subdural hematoma; *SpO2*, oxygen saturation

The incidences of the studied specific conditions were subdural hematoma (SDH) in 26.6%, epidural hematoma (EDH) in 5.4%, cervical spine fractures or ligament injuries in 5.8%, pneumothorax (PTX) in 30.4%, tension pneumothorax in 0.3%, and intraparenchymal brain hemorrhage or ventricular hemorrhage (IPV) in 12.5% of patients.

The median hospital length of stay was 9 days (IQR: 5 to 16). The majority of patients (85.5%) survived their hospitalization, while a 28-day mortality rate of 18.2% was observed.

Additional cohort characteristics are shown in supplemental Table 1 .

### Estimates of prehospital diagnostic accuracy

**Fig. 2:**
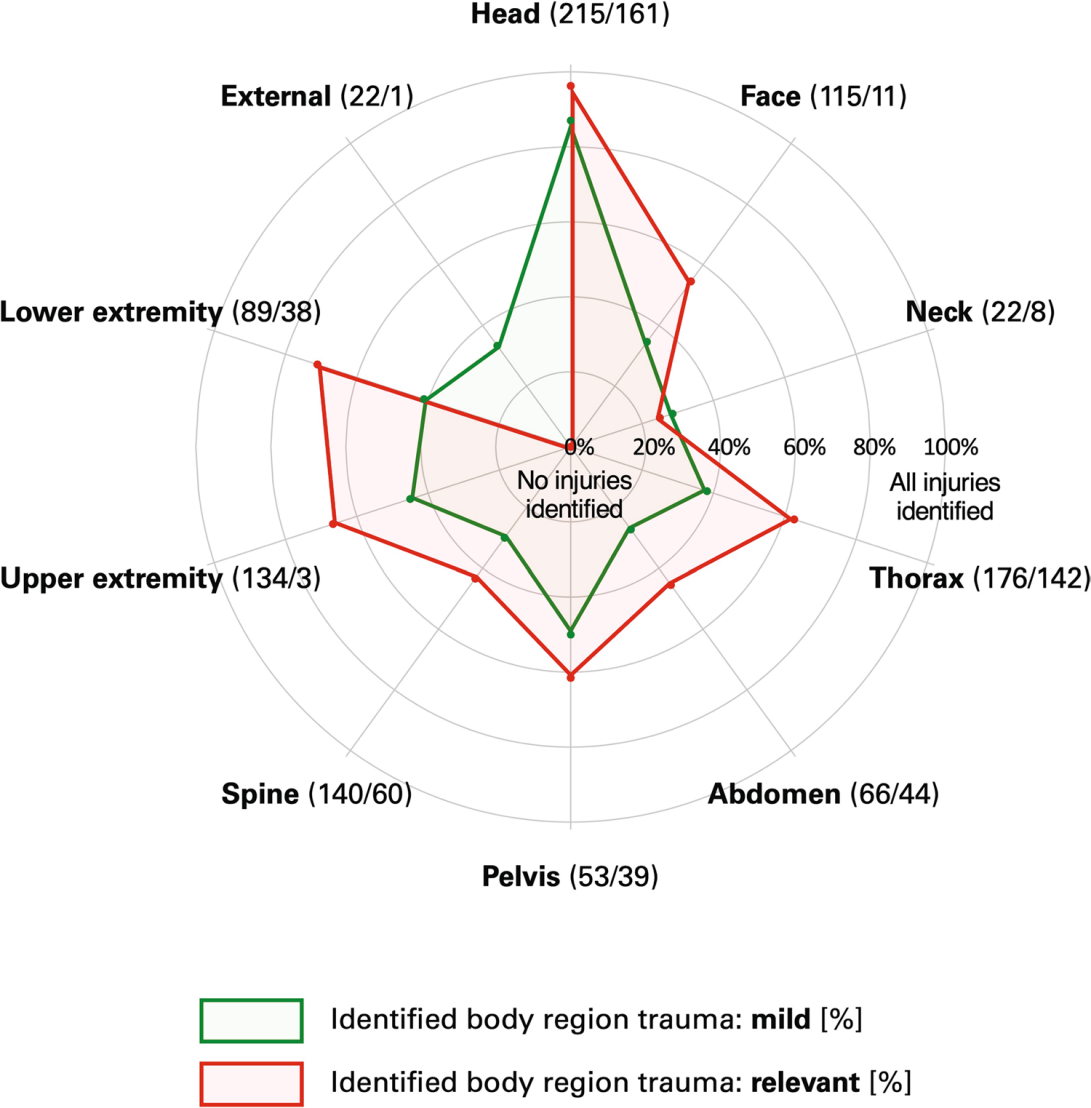
Radar chart of frequency of injuries and percentage of identified injuries according to different body regions and trauma severity. The brackets behind the body region contain the absolute number of mild/relevant injuries in the body region (mild/relevant). The size of the ring corresponds to the percent of prehospital identified injuries

Figure 2 shows the frequency of injuries as well as the percentage of injuries identified prehospital (= sensitivity) according to the different body regions and trauma severity. Relevant head trauma was identified prehospital in 96.3% (95% CI: 92.1%; 98.6%) and relevant chest and pelvis trauma in 62.7% (95% CI: 54.2%; 70.6%) and 61.5% (95% CI: 44.6%; 76.6%) of patients, respectively. Neck (25.0%, 95% CI: 3.19%; 65.1%), spine (43.3%, 95% CI: 30.6%; 56.8%), and abdomen trauma (45.5%, 95% CI: 30.4%; 61.2%) was prehospital less often identified (Table 3 and Supplement Table 2).

**Table 3:** Diagnostic accuracy measures for detecting relevant (AIS ≥ 3) body region injury in the prehospital setting

Body region	Prev	Sens	Spec	PPV	NPV
Head	51.6%	96.3%	47.0%	66.0%	92.2%
Face	3.5%	54.5%	80.4%	9.2%	98.0%
Neck	2.6%	25.0%	85.2%	4.3%	97.7%
Chest	45.5%	62.7%	82.9%	75.4%	72.7%
Abdomen	14.1%	45.5%	88.8%	40.0%	90.8%
Pelvis	12.5%	61.5%	90.1%	47.1%	94.3%
Spine	19.2%	43.3%	82.1%	36.6%	85.9%
Upper extremity	1.0%	66.7%	75.4%	2.6%	99.6%
Lower extremity	12.2%	71.1%	86.9%	42.9%	95.6%
External	0.3%	0.0%	74.6%	0.0%	99.6%
Specific conditions					
SDH	26.6%	97.6%	32.8%	34.5%	97.4%
EDH	5.4%	94.1%	25.8%	6.8%	98.7%
C-spine fracture/ligament injury	5.8%	22.2%	77.2%	5.6%	94.2%
PTX	30.4%	64.2%	73.7%	51.7%	82.5%
Tension PTX	0.3%	100%	62.4%	0.8%	100%
IPV	12.5%	61.5%	90.1%	47.1%	94.3%

*EDH*, epidural hematoma; *IPV*, instable pelvic fracture; *NPV*, negative predictive value; *PPV*, positive predictive value; *Prev*, prevalence; *PTX*, pneumothorax; *SDH*, subdural hematoma; *Sens*, sensitivity; *Spec*, specificity

In all body regions apart from external injuries, severe injuries (AIS ≥ 3) were more likely to be reported preclinical than mild injuries.

The diagnostic accuracy measures with 95% CI for the primary and secondary outcomes are shown in Table 3 as well as supplement Table 2.

The percentage with 95% CI of prehospital not identified/suspected body region trauma as well as the distribution and the accuracy of body region trauma in each AIS severity group is shown in supplement Fig. 1

### Prehospital interventions according to patient’s diagnoses

Overall, 15 of 18 (83.3%) patients with an unstable cervical spine injury received a c-collar; in 22 of 39 (56.4%) patients with an instable pelvic fracture, a pelvic binder was applied. One patient was diagnosed with a tension pneumothorax. This patient received a chest decompression prehospital. Seven out of 95 patients (7.4%) with pneumothorax received a chest decompression in the prehospital setting: four of 24 (16.7%) prehospital intubated patients with pneumothorax were decompressed compared to only 3 of 71 patients (4.2%) who were not prehospitally intubated but had a pneumothorax.

In total, 67.7% without c-spine injury received a cervical collar. A pelvic binder was applied in 19.4% of patients without an unstable pelvic fracture, and in 1.4% of patients without pneumothorax, a chest decompression was performed (see supplement Fig. 2).

## Discussion

In the prehospital setting, it is imperative to identify potentially life-threatening injuries fast and adequately. These injuries may warrant immediate and tailored actions that potentially determine patient’s outcome.

The current study found that severe head trauma was rarely overlooked prehospital, whereas severe chest injuries were frequently unrecognized.

Other high prevalent injuries that remained frequently unrecognized in the prehospital setting were spine, abdominal, and pelvic injuries ranging from 48% for pelvic injuries up to 57% for spine injuries. This is in the nature of things, as the clinical examination in the field is very demanding and often has to take place under adverse conditions, with limited technical equipment and manpower. Despite these challenges in the field, all study patients in the present study were correctly transferred to a level 1 trauma center.

The high sensitivity for suspected head trauma in the prehospital setting of 96.3% is at the expense of a low specificity of only 47%. These findings are in line with a previous publication by Hasler et al. more than 10 years ago [7]. In this retrospective study, HEMS data of 433 patients who were admitted to a single trauma center were evaluated. Consistent with the current study, all patients were prehospitally examined by a physician. The sensitivity for suspected head trauma in the prehospital setting was reported to be 92.9%, similar to our results. These findings are supported by the fact that the level of consciousness and a brief neurologic examination were adequately assessable in the prehospital setting: patients with decreased level of consciousness or any signs of neurologic deficit are considered as potentially brain injured.

Severe chest injuries were present in almost every second patient included here, but remained unrecognized in almost 40% in the prehospital environment. It is well known that it is very challenging to recognize these conditions on scene. Of note, more than 40% of all missions were carried out during late or night shifts with limited light conditions and more than 30% during winter (November to March). Cold or wet weather and additional clothing may further hinder a proper prehospital clinical examination.

The high number of unrecognized prehospital chest injuries in HEMS missions is in line with previous studies [7, 14]. Interestingly, the current study showed that only 7 of 95 patients (7.4%) with pneumothorax received a chest decompression in the prehospital setting. However, it is also important to emphasize that not every pneumothorax requires a prehospital chest decompression. The four times higher chest decompression rate of pneumothoraces in patients who were preclinically intubated compared to those who were not intubated clearly shows the lower threshold for a chest decompression in intubated patients. The more difficult clinical assessment of intubated patients and the fear that high positive pressure during mechanical ventilation could convert a pneumothorax into a tension physiology may explain these findings [15].

However, the overall very low chest decompression rate of patients with a pneumothorax is of concern because any traumatic pneumothorax can progress and lead to tension physiology that has immediate life-threatening consequences, whereas relatively simple prehospital measures such as chest decompression have the potential to resolve this life-threatening situation. Consequently, it appears that patients may have benefitted from more responsive pneumothorax decompression in prehospital HEMS missions. These findings are also supported by a recently published study of our group which compared severely injured patients with those who died prehospitally [13]. In this study, chest trauma was identified in 45% of fatal missions and in almost 30% of severely injured patients. However, pneumothorax decompression was performed in only 17.2% of the fatal cohort and 3.7% in patients who were severely injured.

The lack of diagnostic tools is a major reason for the diagnostic uncertainty in the prehospital setting. A previous study evaluating 255 trauma patients reported that the use of pulse oximetry in addition to physical examination increased the accuracy of the prehospital diagnosis of lung contusion and early detection of tension pneumothorax [16]. Since﻿ 2022, prehospital point-of-care ultrasound has been gradually introduced by Rega HEMS. This tool however, although promising, requires careful evaluation. It is as of yet unclear if prehospital ultrasound leads to improved overall patients’ outcomes, in particular with regard to optimizing prehospital pneumothorax decompression rates [17, 18].

According to the current study, more than 50% of severe abdominal injuries remained unrecognized in prehospital HEMS missions. Similarly, Helm et al. evaluated 479 road traffic accident victims in order to determine the prehospital accuracy [19]. In contrast to the current study, overlooked severe abdominal injuries (AIS ≥ 3) in the prehospital setting were significantly less identified in hypotensive patients with SBP < 90 mmHg (28.6% versus 52.5%, *p* = 0.025). It is possible that existing hypotension in this cohort raised physician’s awareness for the presence of a possible abdominal trauma. In conclusion, measures such as blood pressure monitoring are helpful to increase diagnostic accuracy, and in the absence of other source of bleeding, abdominal hemorrhage should be strongly considered in the presence of hypotension. The increasing implementation of prehospital point-of-care ultrasound may be a promising tool for early identification of abdominal hemorrhage, however, warrants careful evaluation on different outcome levels [17]. Furthermore, it is apparent that hoist rescue (which is usually done in exposed and difficult terrain) hinders preclinical abdominal examination, but should trigger immediate transportation to a level I trauma center.

Another important finding was that almost 40% of pelvic injuries were unrecognized in the prehospital setting. This finding is in line with previous studies [20].

In German-speaking European countries, a pelvic binder is usually placed based on trauma mechanism or clinical findings at the earliest opportunity in order to reduce the risk of serious pelvic hemorrhage [21, 22]. The low detection rate of pelvic injuries may therefore be a main explanation of the low pelvic binder application rate with less than 60% for patients with unstable pelvic fractures. Hasler et al. [7] reported an even worse prehospital detection rate of pelvic injuries with 52%. This lower number compared to our study may be explained by the fact that Hasler et al. included a different patient population with consecutive trauma patients of any severity admitted to a single trauma center. This is reflected by the low mean ISS of 13 compared to the mean ISS of 22 in the current study.

Knowledge of factors associated with overlooked injuries is of paramount importance in order to help triage and trigger fast transportation to a higher level of trauma care. In the study of Wohlgemut et al., unrecognized injury was more common in patients with polytrauma, shock, and uncertainty of the physician [14]. The level of certainty of prehospital diagnoses was classified as certain or uncertain and was based on the Central Intelligence Agency on how humans describe levels of probability [23]. Uncertainty was discussed to be the result of evolving physiology, reduced patient responsiveness (e.g., from head injury or intoxication), or lack of availability of diagnostic adjuncts.

To our knowledge, this is the first multicenter study evaluating diagnostic accuracy in HEMS missions. Our findings identify particular problems with diagnostic accuracy of potentially life-threatening injuries during HEMS missions and may therefore be used for an improvement of prehospital practice. Furthermore, these data help the clinician in the receiving hospital to understand the difficult prehospital conditions and to better interpret and classify the data from the prehospital phase.

It is important to note that the non-HEMS trauma population and the patients attended to by HEMS differ substantially in Switzerland. As demonstrated, patients transported by HEMS are more severely injured compared to those admitted by GEMS. Furthermore, all HEMS come with a dedicated emergency physician, while such a prehospital physician is rare in non-HEMS missions. In addition, the range of operations differs due to the alpine topography of Switzerland. Many severe trauma cases are caused by recreational sports (mountaineering, climbing, paragliding, etc.) and are not accessible by GEMS. The findings presented in the present study are therefore not directly transferable to modes of transportation other than HEMS.

This study has several other limitations. Patients who died prehospitally and those who were not transported to one of the twelve level 1 trauma centers in Switzerland were not included in the present study. A possible selection bias might therefore be present. However, this is likely to be of limited relevance, because only level 1 trauma centers offer the entire spectrum of specialized polytrauma care and therefore have the national mandate to treat these patients. Moreover, many HEMS missions in this analysis involved individuals participating in recreational activities in the mountains during summer and winter (e.g., skiing, hiking, or climbing). Therefore, the findings may not be directly transferable to other countries.

## Conclusions

The difficulties in making correct diagnoses prehospital are considerable. This problem is particularly important for the receiving trauma teams, and a high index of suspicion is required. In addition, patient characteristics that are particularly at risk of missed injuries should be further developed. Especially, urgent chest decompression and measures for consequent hemorrhage control including pelvic binder application are areas for potential improvement in prehospital HEMS missions.

## Supplementary Information

Below is the link to the electronic supplementary material.

Supplementary file1 (EPS 908 KB)

Supplementary file2 (EPS 1.12 MB)

Supplementary file3 (DOCX 13.9 KB)

Supplementary file4 (DOCX 14.3 KB)

Supplementary file5 (DOCX 22 KB)

Supplementary file6 (DOCX 16 KB)

Supplementary file7 (DOCX 17 KB)

## Data Availability

The datasets generated and/or analyzed during the current study are not publicly available, but are available from the corresponding author upon reasonable request.
