# Supplementary material for: Accuracy between prehospital and hospital diagnosis in helicopter emergency medical services and its consequences for trauma care
Source: Eur J Trauma Emerg Surg. 2024 Apr 2;50(4):1681–90. doi: 10.1007/s00068-024-02505-y (PMC11458725; doi:10.1007/s00068-024-02505-y)
Supplement: Supplementary file 3 — Supplementary file3 (DOCX 13.9 KB) [file 68_2024_2505_MOESM3_ESM.docx]

### Supplement 1. Definition for final diagnoses according to AIS codes

- *Subdural hematoma (SDH)* as AIS codes 140438.3 140440.2 140442.4 140446.5 140650.3 140651.3 140652.4 140654.4 140656.5 140655.5
- *Epidural hematoma (EDH)* as AIS codes 140414.3, 140416.2, 140418.4, 140422.5, 140630.3, 140631.2, 140632.4, 140634.5, 140636.5
- *C-Spine fracture/ligament injury* as AIS codes 640284.1, 650204.2, 650206.3, 650216.2, 650234.3,
- *Pneumothorax (PTX)* as AIS codes 442202.2, 442203.4, 442204.5, 442205.3, 442206.4,
- *Tension pneumothorax* as AIS code 442204.5,
- *Instable pelvic fracture (IPF)* as AIS codes 856161.3, 856162.4, 856163.4, 856164.5, 856171.4, 856172.4, 856173.5, 856174.5.
