# Supplementary material for: Accuracy between prehospital and hospital diagnosis in helicopter emergency medical services and its consequences for trauma care
Source: Eur J Trauma Emerg Surg. 2024 Apr 2;50(4):1681–90. doi: 10.1007/s00068-024-02505-y (PMC11458725; doi:10.1007/s00068-024-02505-y)
Supplement: Supplementary file 4 — Supplementary file4 (DOCX 14.3 KB) [file 68_2024_2505_MOESM4_ESM.docx]

### Supplement 2. Detailed information of excluded variables

- *Demographics (STR):* age and gender
- *Rescue characteristics (mission protocol):* shift, response time, duration flight, on scene time and if a hoist was used for the rescue
- *Accident characteristics (STR):* type of injury in particular penetrating trauma, traffic - car, truck, traffic - motor cycle crash, bicycle as well as falls (over 3m, below 3m, unknown height)
- *First measured vital signs (prehospital from the mission protocol and clinical from STR):* Glascow coma scale (GCS), heart rate (HR), systolic blood pressure (SBP) and oxygen saturation (SpO2) as well as additionally from the STR the first respiratory rate, and temperature.
- *Prehospital actions (mission protocol):* describes in detail elsewhere, in particular usage of neck collars or pelvic belts, airway management techniques
- *Injury severity:* the injury severity score (STR) and estimated injury severity with NACA score (mission protocol)
- *Clinical outcome (STR):* Duration of hospitalization, survival of hospitalization and 28days-mortality
