# Supplementary material for: Accuracy between prehospital and hospital diagnosis in helicopter emergency medical services and its consequences for trauma care
Source: Eur J Trauma Emerg Surg. 2024 Apr 2;50(4):1681–90. doi: 10.1007/s00068-024-02505-y (PMC11458725; doi:10.1007/s00068-024-02505-y)
Supplement: Supplementary file 5 — Supplementary file5 (DOCX 22 KB) [file 68_2024_2505_MOESM5_ESM.docx]

|  | | | **N** | **Total** | **(n=312)** |
| --- | --- | --- | --- | --- | --- |
| **RESCUE AND ACCIDENT CHARACTERISTICS** | | |  |  |  |
| **Shift, n (%)** | | | 312 |  |  |
| Day shift (8-16) | |  | 184 | [59.0] | |
| Late shift (16-24) | |  | 95 | [30.4] | |
| Night shift (24-8) | |  | 33 | [10.6] | |
| **Months of operation, n (%)** | | | 312 |  |  |
| January | | |  | 16 | [5.1] |
| February | | |  | 18 | [5.8] |
| March | | |  | 25 | [8.0] |
| April | | |  | 20 | [6.4] |
| May | | |  | 31 | [9.9] |
| June | | |  | 24 | [7.7] |
| July | | |  | 49 | [15.7] |
| August | | |  | 30 | [9.6] |
| September | | |  | 35 | [11.2] |
| October | | |  | 21 | [6.7] |
| November | | |  | 26 | [8.3] |
| December | | |  | 17 | [5.4] |
| **Type of injury, detail, n (%)** | | | 312 |  |  |
| Traffic - car, truck | | |  | 24 | [7.7] |
| Traffic - motor cycle crash | | |  | 33 | [10.6] |
| Bicycle | | |  | 35 | [11.2] |
| Pedestrian | | |  | 12 | [3.8] |
| Fall over 3m | | |  | 77 | [24.7] |
| Fall below 3m | | |  | 58 | [18.6] |
| Fall of unknown height | | |  | 12 | [3.8] |
| Traffic other | | |  | 8 | [2.6] |
| Other - Blow | | |  | 16 | [5.1] |
| Gunshot wound | | |  | 1 | [0.3] |
| Stab wound | | |  | 1 | [0.3] |
| Other | | |  | 32 | [10.3] |
| Avalanche, landslide, spillage | | |  | 1 | [0.3] |
| Unknown | | |  | 2 | [0.6] |
| **Penetrating trauma, n (%)** | | 312 | | 24 | [7.7] |
| **Hoist rescue, n (%)** | | 312 | | 49 | [15.7] |
| **INJURY CHARACTERISTICS** | | |  |  |  |
| **AIS head, n (%)** | | | 312 |  |  |
| 0 | | |  | 97 | [31.1] |
| 1 | | |  | 23 | [7.4] |
| 2 | | |  | 31 | [9.9] |
| 3 | | |  | 66 | [21.2] |
| 4 | | |  | 45 | [14.4] |
| 5 | | |  | 50 | [16.0] |
| **AIS face, n (%)** | | | 312 |  |  |
| 0 | | |  | 197 | [63.1] |
| 1 | | |  | 51 | [16.3] |
| 2 | | |  | 53 | [17.0] |
| 3 | | |  | 11 | [3.5] |
| **AIS neck, n (%)** | | | 312 |  |  |
| 0 | | |  | 290 | [92.9] |
| 1 | | |  | 3 | [1.0] |
| 2 | | |  | 11 | [3.5] |
| 3 | | |  | 6 | [1.9] |
| 4 | | |  | 2 | [0.6] |
| **AIS thorax, n (%)** | | | 312 |  |  |
| 0 | | |  | 136 | [43.6] |
| 1 | | |  | 12 | [3.8] |
| 2 | | |  | 22 | [7.1] |
| 3 | | |  | 108 | [34.6] |
| 4 | | |  | 27 | [8.7] |
| 5 | | |  | 7 | [2.2] |
| **AIS abdomen, n (%)** | | | 312 |  |  |
| 0 | | |  | 246 | [78.8] |
| 1 | | |  | 7 | [2.2] |
| 2 | | |  | 15 | [4.8] |
| 3 | | |  | 19 | [6.1] |
| 4 | | |  | 19 | [6.1] |
| 5 | | |  | 6 | [1.9] |
| **AIS spine, n (%)** | | | 312 |  |  |
| 0 | | |  | 172 | [55.1] |
| 1 | | |  | 4 | [1.3] |
| 2 | | |  | 76 | [24.4] |
| 3 | | |  | 32 | [10.3] |
| 4 | | |  | 8 | [2.6] |
| 5 | | |  | 19 | [6.1] |
| 6 | | |  | 1 | [0.3] |
| **AIS upper extremity, n (%)** | | | 312 |  |  |
| 0 | | |  | 178 | [57.1] |
| 1 | | |  | 22 | [7.1] |
| 2 | | |  | 109 | [34.9] |
| 3 | | |  | 3 | [1.0] |
| **AIS lower extremity (excl. pelvis), n (%)** | | | 312 |  |  |
| 0 | | |  | 223 | [71.5] |
| 1 | | |  | 24 | [7.7] |
| 2 | | |  | 27 | [8.7] |
| 3 | | |  | 37 | [11.9] |
| 4 | | |  | 1 | [0.3] |
| **AIS pelvis only, n (%)** | | | 312 |  |  |
| 0 | | |  | 259 | [83.0] |
| 2 | | |  | 14 | [4.5] |
| 3 | | |  | 9 | [2.9] |
| 4 | | |  | 22 | [7.1] |
| 5 | | |  | 8 | [2.6] |
| **AIS lower extremity (incl. pelvis), n (%)** | | | 312 |  |  |
| 0 | | |  | 198 | [63.5] |
| 1 | | |  | 18 | [5.8] |
| 2 | | |  | 28 | [9.0] |
| 3 | | |  | 37 | [11.9] |
| 4 | | |  | 23 | [7.4] |
| 5 | | |  | 8 | [2.6] |
| **AIS external, n (%)** | | | 312 |  |  |
| 0 | | |  | 290 | [92.9] |
| 1 | | |  | 21 | [6.7] |
| 4 | | |  | 1 | [0.3] |
| **COHORT AND CLINICAL CHARACTERISTICS** | | |  |  |  |
| Age >65 years, n (%) | | | 312 | 90 | [28.8] |
| Gender, n (%) | | | 312 |  |  |
| Male | | |  | 241 | [77.2] |
| Female | | |  | 71 | [22.8] |
| NACA score, mean (SD) | | | 312 | 4.4 | [0.6] |
| GCS, median (IQR) | | | 312 | 14 | [7; 15] |
| GCS 1st, prehospital <9, n (%) | | | 312 | 91 | [29.2] |
| HR 1st, prehospital [bpm], median (IQR) | | | 295 | 85 | [70; 103] |
| HR 1st, prehospital >120/min, n (%) | | | 295 | 18 | [6.1] |
| SBP 1st, prehospital [mmHg], mean (SD) | | | 254 | 127 | [29.0] |
| SBP 1st, prehospital <100mmHg, n (%) | | | 254 | 38 | [15.0] |
| SpO2 1st, prehospital [%], median (IQR) | | | 285 | 96 | [91; 98] |
| SpO2 1st, prehospital <90%, n (%) | | | 285 | 45 | [15.8] |
