# Supplementary material for: Accuracy between prehospital and hospital diagnosis in helicopter emergency medical services and its consequences for trauma care
Source: Eur J Trauma Emerg Surg. 2024 Apr 2;50(4):1681–90. doi: 10.1007/s00068-024-02505-y (PMC11458725; doi:10.1007/s00068-024-02505-y)
Supplement: Supplementary file 6 — Supplementary file6 (DOCX 16 KB) [file 68_2024_2505_MOESM6_ESM.docx]

|  | **Sensitivity** | | **Specificity** | | **Positive predictive value** | | **Negative predictive value** | |
| --- | --- | --- | --- | --- | --- | --- | --- | --- |
| **Head** | 96.3% | (95% CI: 92.1%; 98.6%) | 47% | (95% CI: 38.9%; 55.3%) | 66% | (95% CI: 59.5%; 72%) | 92.2% | (95% CI: 83.8%; 97.1%) |
| **Face** | 54.5% | (95% CI: 23.4%; 83.3%) | 80.4% | (95% CI: 75.5%; 84.7%) | 9.2% | (95% CI: 3.5%; 19%) | 98.0% | (95% CI: 95.3%; 99.3%) |
| **Neck** | 25.0% | (95% CI: 3.19%; 65.1%) | 85.2% | (95% CI: 80.7%; 89%) | 4.3% | (95% CI: 0.5%; 14.5%) | 97.7% | (95% CI: 95.1%; 99.2%) |
| **Chest** | 62.7% | (95% CI: 54.2%; 70.6%) | 82.9% | (95% CI: 76.4%; 88.3%) | 75.4% | (95% CI: 66.6%; 82.9%) | 72.7% | (95% CI: 65.8%; 78.8%) |
| **Abdomen** | 45.5% | (95% CI: 30.4%; 61.2%) | 88.8% | (95% CI: 84.4%; 92.3%) | 40.0% | (95% CI: 26.4%; 54.8%) | 90.8% | (95% CI: 86.7%; 94%) |
| **Pelvis** | 61.5% | (95% CI: 44.6%; 76.6%) | 90.1% | (95% CI: 85.9%; 93.4%) | 47.1% | (95% CI: 32.9%; 61.5%) | 94.3% | (95% CI: 90.7%; 96.7%) |
| **Spine** | 43.3% | (95% CI: 30.6%; 56.8%) | 82.1% | (95% CI: 76.8%; 86.7%) | 36.6% | (95% CI: 25.5%; 48.9%) | 85.9% | (95% CI: 80.8%; 90%) |
| **Upper extremity** | 66.7% | (95% CI: 9.43%; 99.2%) | 75.4% | (95% CI: 70.2%; 80.1%) | 2.6% | (95% CI: 0.3%; 9.0%) | 99.6% | (95% CI: 97.6%; 100%) |
| **Lower extremity** | 71.1% | (95% CI: 54.1%; 84.6%) | 86.9% | (95% CI: 82.3%; 90.6%) | 42.9% | (95% CI: 30.5%; 56%) | 95.6% | (95% CI: 92.2%; 97.8%) |
| **External** | 0% | (95% CI: 0%; 97.5%) | 74.6% | (95% CI: 69.4%; 79.3%) | 0% | (95% CI: 0%; 4.6%) | 99.6% | (95% CI: 97.6%; 100%) |
|  |  |  |  |  |  |  |  |  |
| **Specific conditions** | | |  |  |  |  |  |  |
| **SDH** | 97.6% | (95% CI: 91.6%; 99.7%) | 32.8% | (95% CI: 26.7%; 39.2%) | 34.5% | (95% CI: 28.4%; 40.9%) | 97.4% | (95% CI: 90.9%; 99.7%) |
| **EDH** | 94.1% | (95% CI: 71.3%; 99.9%) | 25.8% | (95% CI: 20.9%; 31.2%) | 6.81% | (95% CI: 3.94%; 10.8%) | 98.7% | (95% CI: 93%; 100%) |
| **C-spine** | 5.6% | (95% CI: 1.6%; 13.8%) | 94.2% | (95% CI: 90.4%; 96.8%) | 22.2% | (95% CI: 6.4%; 47.6%) | 77.2% | (95% CI: 72%; 81.9%) |
| **PTX** | 64.2% | (95% CI: 53.7%; 73.8%) | 73.7% | (95% CI: 67.3%; 79.5%) | 51.7% | (95% CI: 42.3%; 61%) | 82.5% | (95% CI: 76.4%; 87.5%) |
| **Tension PTX** | 100% | (95% CI: 2.5%; 100%) | 62.4% | (95% CI: 56.7%; 67.8%) | .8% | (95% CI: 0.0%; 4.6%) | 100% | (95% CI: 98.1%; 100%) |
| **IPV** | 61.5% | (95% CI: 44.6%; 76.6%) | 90.1% | (95% CI: 85.9%; 93.4%) | 47.1% | (95% CI: 32.9%; 61.5%) | 94.3% | (95% CI: 90.7%; 96.7%) |
