# Supplementary material for: Accuracy between prehospital and hospital diagnosis in helicopter emergency medical services and its consequences for trauma care
Source: Eur J Trauma Emerg Surg. 2024 Apr 2;50(4):1681–90. doi: 10.1007/s00068-024-02505-y (PMC11458725; doi:10.1007/s00068-024-02505-y)
Supplement: Supplementary file 7 — Supplementary file7 (DOCX 17 KB) [file 68_2024_2505_MOESM7_ESM.docx]

|  | **GEMS*** | **(n=1316)** | **Study collective** | **(n=312)** |
| --- | --- | --- | --- | --- |
|  |  |  |  |  |
| **DEMOGRAPHICS** |  |  |  |  |
| **Age [years], median (IQR)** | 67 | [48; 81] | 54.5 | [34; 68] |
| **Gender, n (%)** |  |  |  |  |
| Female | 469 | [35.6] | 75 | [24.0] |
| Male | 847 | [64.4] | 237 | [76.0] |
| **VITALS, 1^st^ CLINICAL** |  |  |  |  |
| SBP [mmHg] , median (IQR) | 138.0 | [120.0; 160.0] | 130.5 | [113.5; 150.0] |
| Pulse [bpm], median (IQR) | 82 | [71; 95] | 86 | [72; 98] |
| GCS, median (IQR) | 14 | [12; 15] | 14 | [3; 15] |
| Respiratory rate [/min], median (IQR) | 18 | [15; 21] | 17 | [14; 21] |
| Temperature[°C], median (IQR) | 36.5 | [36.0; 37.0] | 36.4 | [35.9; 36.8] |
| SpO2 [%], median (IQR) | 97 | [95; 99] | 98 | [95; 100] |
| **INJURY CHARACTERISTICS & RELEVANT TRAUMA** |  |  |  |  |
| ISS, median (IQR) | 18 | [14; 25] | 22 | [17; 29] |
| Head, n (%) | 1002 | [76.1] | 161 | [51.6] |
| Face, n (%) | 27 | [2.1] | 11 | [3.5] |
| Neck, n (%) | 14 | [1.1] | 8 | [2.6] |
| Thorax, n (%) | 330 | [25.1] | 142 | [45.5] |
| Abdomen, n (%) | 99 | [7.5] | 44 | [14.1] |
| Pelvis, n (%) | 96 | [7.3] | 39 | [12.5] |
| Spine, n (%) | 90 | [6.8] | 60 | [19.2] |
| Upper extremity, n (%) | 8 | [0.6] | 3 | [1.0] |
| Lower extremity, n (%) | 161 | [12.2] | 38 | [12.2] |
| **SPECIFIC CONDITIONS** |  |  |  |  |
| SDH, n (%) | 736 | [55.9] | 83 | [26.6] |
| EDH, n (%) | 134 | [10.2] | 17 | [5.4] |
| C-spine fracture/ligament injury, n (%) | 38 | [2.9] | 18 | [5.8] |
| PTX, n (%) | 199 | [15.1] | 95 | [30.4] |
| Tension PTX, n (%) | 6 | [0.5] | 1 | [0.3] |
| IPV, n (%) | 96 | [7.3] | 39 | [12.5] |
| **OUTCOME** |  |  |  |  |
| Duration of hospitalization [days], median (IQR) | 8 | [4; 13] | 9 | [5; 16] |
| Survival hospitalisation, n (%) | 1153 | [87.6] | 266 | [85.5] |
| Mortality 28 days, n (%) | 185 | [15.9] | 49 | [18.2] |
